# Calcium silicate-stimulated adipose-derived stem cells promote angiogenesis and improve skin wound healing

**DOI:** 10.18632/aging.204760

**Published:** 2023-06-01

**Authors:** Mingming Wang, Hongyan Zhan, Jianhua Wang, Hua Song, Jianhua Sun, Gang Zhao

**Affiliations:** 1 Department of Orthopaedics, Jinan Central Hospital, Cheeloo College of Medicine, Shandong University, Jinan, Shandong, China; 2 Department of B-Ultrasound, The Fourth People’s Hospital of Jinan, Jinan, Shandong, China; 3 Department of Orthopaedics, Tengzhou Central People’s Hospital, Tengzhou, Shandong, China

**Keywords:** wound healing, ADSCs, calcium silicate, HUVEC, CXCR4

## Abstract

Skin wound healing is a complicated process involving proliferation, inflammation, coagulation, and hemostasis, and scar tissue formation of wound repairing. Adipose-derived stem cells (ADSCs) have presented potential therapeutic effects in the non-healing and chronic wound. Calcium silicate (CS) ceramics have been identified as a new type of bioceramics for tissue construction and regeneration. Here, we aimed to explore the impact of CS on the regulation of ADSCs-mediated wound healing. Significantly, CS was able to dose-dependently enhance the proliferation of ADSCs. CS inhibited terminal deoxynucleotidyl transferase dUTP nick end labeling positive cells in the H_2_O_2_-treated ADSCs. Similarly, the Bcl-2 expression was elevated while Bax and cleaved caspase-3 expression were repressed by CS in the cells. CS could induce migration and reduce oxidative stress of ADSCs. Moreover, immunofluorescence analysis and Western blot analysis showed that CS could promote CXCR4 expression in ADSCs. Moreover, CS-stimulated ADSCs enhanced migration and angiogenic capacity of HUVEC. Importantly, CS-stimulated ADSCs improved wound healing in full-thickness skin defect mouse model. Thus, we conclude that CS improves ADSCs-attenuated wound healing *in vivo* and *in vitro*. Our finding presents novel insight in the scenario that CS regulates ADSCs and wound healing. CS may be applied as potential materials for the treatment of wound healing.

## INTRODUCTION

Wound healing serves as an exceptionally continuous and complex involving proliferation, inflammation, coagulation, and hemostasis, and scar tissue formation of wound repairing [1, 2]. Improper wound care management increases the potential for complications in healing, including non-healing or a persisting wound [3, 4]. The therapeutic strategies of improving the skin wound healing are limited and urgently needed [5, 6]. As a stem cell-based therapy, adipose-derived stem cells (ADSCs) have been identified to induce tissue regeneration in the non-healing and chronic wound because of paracrine effects and differentiation [7]. Previous studies reveal that ADSCs present the capability of regulating neovascularization and angiogenesis [8–10]. ADSCs are able to discharge multiple bioactive factors, containing hepatocyte growth factor (HGF), epidermal growth factor (EGF), and vascular endothelial growth factor (VEGF), which benefit promoting angiogenesis and enhancing endothelial cell (EC) function [11]. ADSCs release extracellular vesicles (EVs) and can induce a pro-angiogenic role in promoting the proliferation of human microvascular endothelial cells (HMECs) [12]. Moreover, ADSCs increase flap survival through enhancing blood perfusion and promoting angiogenic response in epigastric artery skin flap in the rat model [13]. In addition, remarkably stimulated neovascularization has been observed in the rabbit model’s venous congested skin graft treated with ADSCs [14].

In recent years, calcium silicate (CS) ceramics are identified as possibly a new sort of bioceramics for bone tissue construction employment and bone regeneration [15]. Previous studies have confirmed that CS presents biodegradability and superior bioactivity in the bioceramics possess [16]. Meanwhile, it has been found that CS well-supports the proliferation and attachment of osteoblasts and bone marrow mesenchymal stem cells [17], The bioactive Si ions from CS provides an excellent extracellular setting of pointing BMSCs differentiation and proliferation for the osteogenic progenitors [18], and promoting the angiogenesis and proliferation development of the human umbilical vein endothelial cells (HUVECs) [19]. CS enhances supplementary bone formation compared with traditionally applied bioceramics of β-tricalcium phosphate (β-TCP) [20]. Moreover, no-acute inflammation response has been shown at the interaction of CS and host tissues [21]. However, the function of CS in the modulation of ADSCs and wound healing is still unreported.

In this study, we were interested in the role of CS in the regulation ofADSCs-mediated wound healing *in vivo* and *in vitro*. We identified a novel function of CS in improving ADSCs-attenuated wound healing.

## MATERIALS AND METHODS

### Cell isolation and treatment

ADSCs isolation was carried out as described [22]. The subcutaneous fats were obtained from the Balb/c mice (12-weeks old, male). The phosphate-buffered saline (PBS, Gibco, USA) containing penicillin-streptomycin (1%, Sigma, USA) was used to wash the adipose, followed by the plating into pieces (<1 mm) and centrifuge (400 g, 5 minutes). For the floating tissue, the collagenase I (1%, Gibco, USA) was placed in the floating tissues and digested in the water bath (37°C), and gently rocked for 45 minutes every 5 minutes. The undigested tissues were removed by a 70-μm filter. The cells were cultured at the incubator of 5% CO_2_ and 37°C with low-glucose DMEM medium. The ADSCs were treated with H_2_O_2_ (50 mM) for 48 hours for the hypoxia stimulation. CS was purchased (Sigma, USA), and the ADSCs were treated with CS at the indicated doses. HUVEC were cultured in DMEM (Gibco, USA) with 10% FBS (Gibco, USA). The co-culture of ADSCs and HUVEC was performed *in vitro* as previously described [23].

### Wound healing mouse model

The wound healing mouse model was established as the previous report [24]. Balb/c mice (8-weeks old, 22 ± 4 g, male) were applied in this study. Before the operational scheme, the mice intraperitoneally received anesthesia of sodium pentobarbital (50 mg/kg). Their dorsal exterior, consisting of the surgical region, was exhaustively stripped and washed with ethyl alcohol (75%) three times. The mice were randomly divided into 5 groups (*n* = 5) according to the different treatment after surgery. Two-round shapes of the full-thick-ness wound were conducted. To assess the wound healing, the photograph of the wounded area was recorded by the digital camera. Animal care and method procedure were authorized by the Animal Ethics Committee of Jinan Central Hospital.

### Biochemistry analysis

The cellular ROS production was analyzed using 7′-Dichlorodihydrofluorescein Diacetate (DCFH-DA) staining (Jiancheng, China) according to the manufacturer's instruction. The NO levels were analyzed using the NO detection Kit (Solarbio, China). The SOD activities were analyzed using the SOD detection Kit (Solarbio, China).

### CCK-8 assays

The proliferation was assessed using CCK-8 assays. About 1 × 10^3^ cells were plated in 96-well dishes and incubated for the transfection or treatment. AThe cells were added with a CCK-8 solution (KeyGEN Biotech, China) and culture for another 2 hours at 37°C. The proliferation was measured at a absorbance of 450 nm by applying the ELISA browser (EL 800, Bio-Tek, USA).

### Terminal deoxynucleotidyl transferase dUTP nick end labeling (TUNEL)

The apoptosis was analyzed by using the TUNEL detection kit (Roche, Germany) according to the product’s guidance. After the staining of TUNEL, the ventricular samples were dyed by DAPI (Sigma, USA) to stain nuclear. Fluorescence was observed using a confocal microscope (Olympus Fluoview1000, Tokyo, Japan).

### Tube formation assays

The angiogenic capacity was analyzed by tube formation assays (BD Biosciences, USA). The HUVEC was plated in 24-well dishes and incubated at 37°C for 24 hours. After that, tube formation was performed by microscopy.

### Western blot analysis

RIPA buffer (CST, USA) was used to extract the total protein, followed by the quantification based on the BCA method (Abbkine, USA). The proteins at same concentration were subjected in SDS-PAGE and transferred (PVDF, Millipore, USA), followed by the incubation with 5% milk and with the primary antibodies at 4°C overnight. The corresponding second antibodies (Boster, China) were used for incubating the membranes 1 hour at room temperature, followed by the visualization by using chemiluminescence detection kit (Beyyotime, China). The primary antibodies applied in this study comprising Bax (1:2000, ab32502, Abcam, USA), Bcl-2 (1:2000, ab32124, Abcam, USA), caspase-3 (1:2000, ab32351, Abcam, USA), and β-actin (1:2000, ab 8226, Abcam, USA). The grayscale of the images was quantified and calculated using a gel image system (Bio-Rad, USA) and the relative level of each band was normalized to the level of β-actin.

### Transwell assays

Transwell assays analyzed the migration of ADSCs and HUVEC by using a Transwell plate (Corning, USA) according to the manufacturer’s instruction. Briefly, the upper chambers were plated with around 1 × 10^5^ cells. Then solidified through 4% paraformaldehyde and dyed with crystal violet. The invaded and migrated cells were recorded and calculated.

### Immunofluorescence analysis

Cells were solidified at 4% paraformaldehyde for 30 min, treated with Triton X 100 (0.2%) for 10 min and treated with BSA (2%) for 30 minutes. The slides were hatched with the primary antibody overnight at 4°C, then hatched with secondary antibodies (Proteintech, China) for 1 hour at 37°C. The slides were stained with the Hoechst (Beyotime, China) for 10 min at 25°C. The Nikon microscope (Tokyo, Japan) was utilized to analyze the immunofluorescence.

### Histological and immunohistochemical analyses

The slices of skin tissues (5 μm thick) were stained with Masson’s trichrome. The immunohistochemical staining in heart sections was performed by using the primary antibody. Heat-induced epitope retrieval was performed with tris-EDTA buffer (pH 9.0) at 110°C for 12 min. The photographs were captured by an Olympus BX60 microscope (Olympus Optical, Tokyo, Japan) at a magnification ×200.

### Quantitative PCR (qPCR)

Total RNA was isolated using TRIzol Solarbio, China) and the first-strand cDNA was manufactured (TaKaRa, China). The qPCR was carried out *via* applying SYBR-Green (Takara, China). The primer sequences are as follows:

**Table:** 

VEGF sense	5′-TCACCAAGGCCAGCACATAG-3′
VEGF anti-sense	5′-GAGGCTCCAGGGCATTAGAC-3′
VEGFR2 sense	5′-CGTCAACAAAGTCGGGAGA-3′
VEGFR2 anti-sense	5′-CAGTGCACCACAAAGACACG-3′
bFGF sense	5′-CGGTCAACAAAGTCGGGAGA-3′
bFGF anti-sense	5′-CAGTGCACCACAAAGACACG-3′
bFGFR sense	5′-CTGGTGATGATGGTGAAG-3′
bFGFR anti-sense	5′-CCTGGATAACCTCTGTGA-3′
GAPDH sense	5′-TATGATGATATCAAGAGGGTAGT-3′
GAPDH anti-sense	5′-TATGATGATATCAAGAGGGTAGT-3′

### Statistical analysis

Data were presented as mean ± SEM, and the statistical analysis was performed by SPSS software (version 18.0, SPSS Inc., USA). The unpaired Student’s *t*-test was applied for comparing two groups, and the one-way ANOVA was applied for comparing among multiple groups. When the data do not conform to the normal distribution, the Mann–Whitney *U* test was used for comparisons between two groups and the Kruskal–Wallis test with Dunn’s multiple comparisons post-test was used for comparisons among multiple groups. *P* < 0.05 were considered as statistically significant.

### Data availability statement

No data were used to support this study.

## RESULTS

### CS enhances the proliferation of ADSCs

**Figure 1:**
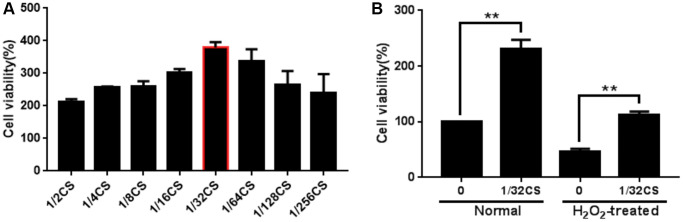
**CS enhances the proliferation of ADSCs.** (**A**, **B**) The ADSCs were treated with at the indicated doses. (**A**) The cell proliferation was analyzed by CCK-8 assays in the cells. (**B**) The H_2_O_2_-treated ADSCs were treated with CS at the indicated doses. The cell proliferation was assessed by CCK-8 assays in the cells. Data are presented as mean ± SD. Statistic significant differences were indicated: ^*^P < 0.05, ^**^P < 0.01.

To evaluate the potential impact of CS on the regulation of ADSCs, the ADSCs were treated with CS as the indicated concentrations. Significantly, CCK-8 assays showed that the treatment of CS dose-dependently enhanced the viability of ADSCs (Figure 1A). Moreover, the cell proliferation of H_2_O_2_-treated ADSCs was promoted by CS in a dose-dependent manner, in which 1/32 CS presented the highest activities and was used in the subsequent experiments (Figure 1B). Together these data indicate that CS is able to enhance the proliferation of ADSCs.

### CS inhibits the apoptosis of ADSCs

**Figure 2:**
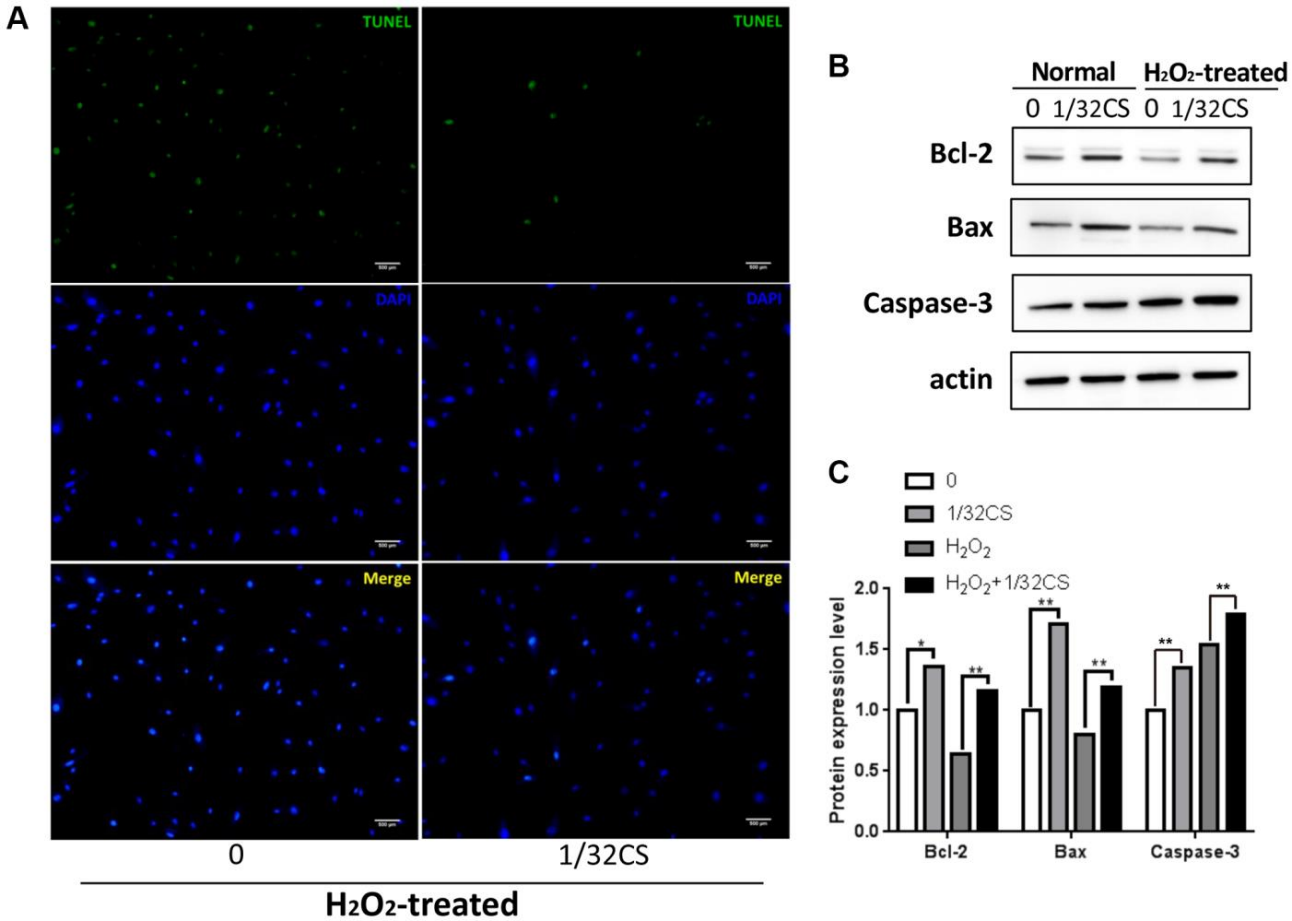
**CS inhibits the apoptosis of ADSCs.** (**A**–**C**) The H_2_O_2_-treated ADSCs were treated with CS at the indicated doses. (**A**) The apoptosis was determined by TUNEL assays in the cells. (**B**, **C**) The expression levels of Bax, Bcl-2, caspase-3, and β-actin were determined by Western blot analysis in the cells. The results of Western blot analysis were quantified by ImageJ software. Data are presented as mean ± SD. Statistic significant differences were indicated: ^*^P < 0.05, ^**^P < 0.01.

Next, we analyzed the function of CS in the modulation of apoptosis of ADSCs. We observed that the treatment of CS significantly decreased TUNEL positive cells in the H_2_O_2_-treated ADSCs (Figure 2A). Similarly, the Bcl-2 expression was up-regulated while the Bax and cleaved caspase-3 expression were down-regulated by CS in the cells (Figure 2B, 2C), suggesting that CS can inhibit the apoptosis of ADSCs.

### CS induces migration and inhibits oxidative stress of ADSCs

**Figure 3:**
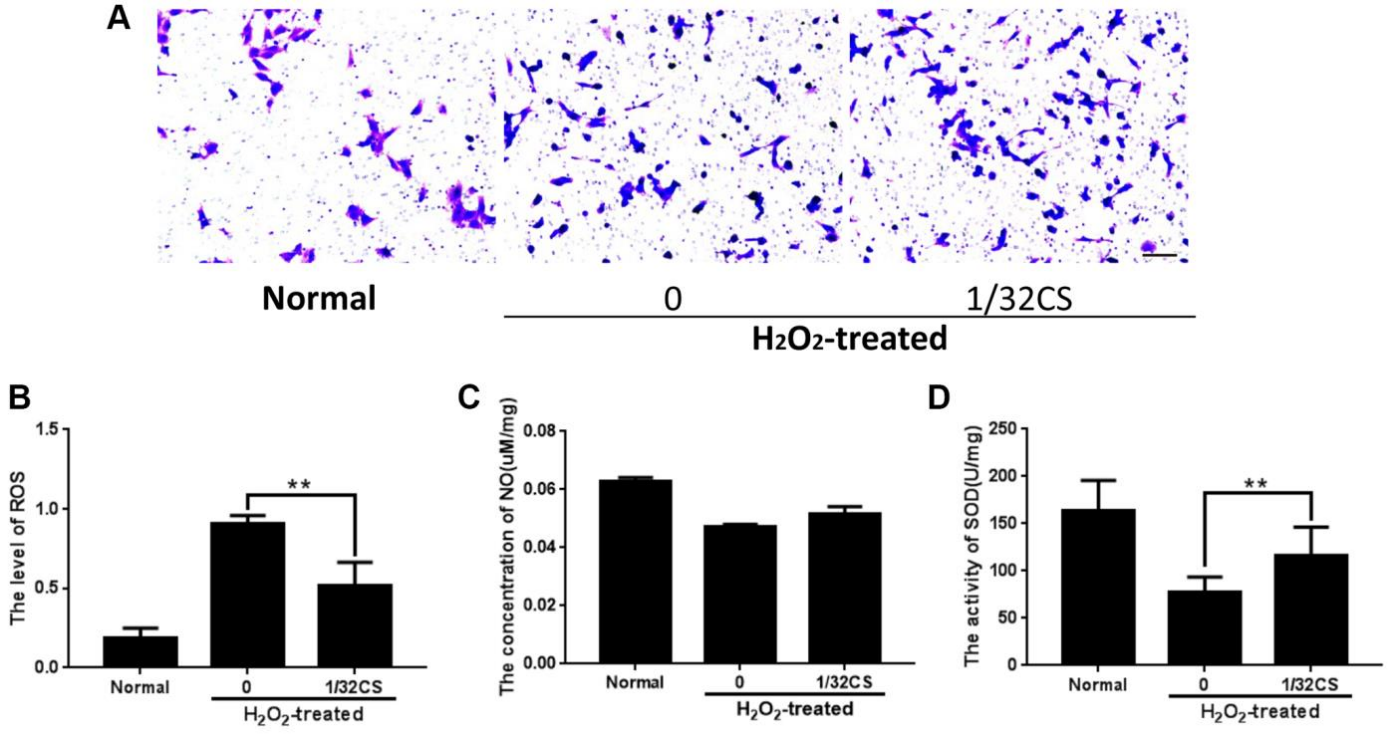
**CS induces migration and inhibits oxidative stress of ADSCs.** (**A**–**D**) The H_2_O_2_-treated ADSCs were treated with CS at the indicated doses. (**A**) The migration was tested by transwell assays in the cells. (**B**–**D**) The levels of ROS, NO, and SOD were examined by the corresponding measurement kit in the cells. Scal bar: 20 μm. Data are presented as mean ± SD. Statistic significant differences were indicated: ^*^P < 0.05, ^**^P < 0.01.

Then, we were interested in the effect of CS on the migration and oxidative stress of ADSCs. Trasnwell assays revealed that the treatment of CS remarkably induced migration of ADSCs (Figure 3A). Meanwhile, the treatment of H_2_O_2_ reduced the levels of SOD and NO but enhanced the ROS levels in the ADSCs, in which the treatment of CS could reverse this effect in the system (Figure 3B–3D). Taken together, these data indicate that CS is able to induce migration and inhibit oxidative stress of ADSCs.

### CS increases CXCR4 expression in ADSCs

**Figure 4:**
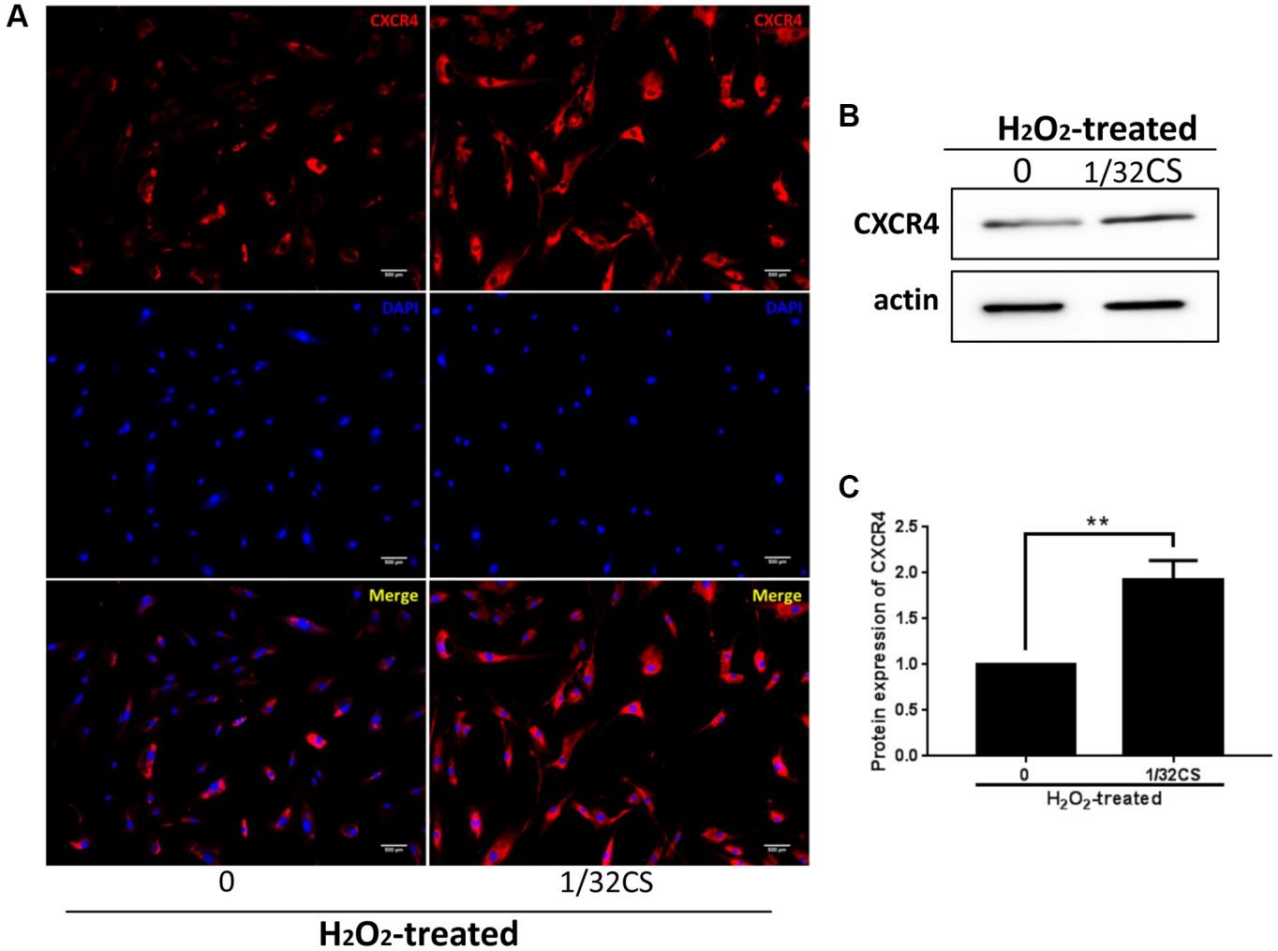
**CS increases CXCR4 expression in ADSCs.** (**A**–**C**) The H_2_O_2_-treated ADSCs were treated with CS at the indicated doses. (**A**) The expression of CXCR4 was measured by immunofluorescence analysis in the cells. (**B**, **C**) The levels of CXCR4 were assessed by Western blot analysis in the cells. The results of Western blot analysis were quantified by ImageJ software. Data are presented as mean ± SD. Statistic significant differences were indicated: ^*^P < 0.05, ^**^P < 0.01.

Given that CXCR4 exerted a critical role in the modulation of ADSCs, we then explored whether CS regulated ADSCs by mediating CXCR4. Significantly, immunofluorescence analysis showed that the treatment of CS was able to increase the CXCR4 accumulation in the ADSCs (Figure 4A). In addition, Western blot analysis presented a similar result in the cells (Figure 4B, 4C), indicating that CS can promote CXCR4 expression in ADSCs.

### CS-stimulated ADSCs enhance migration and angiogenic capacity of HUVEC

**Figure 5:**
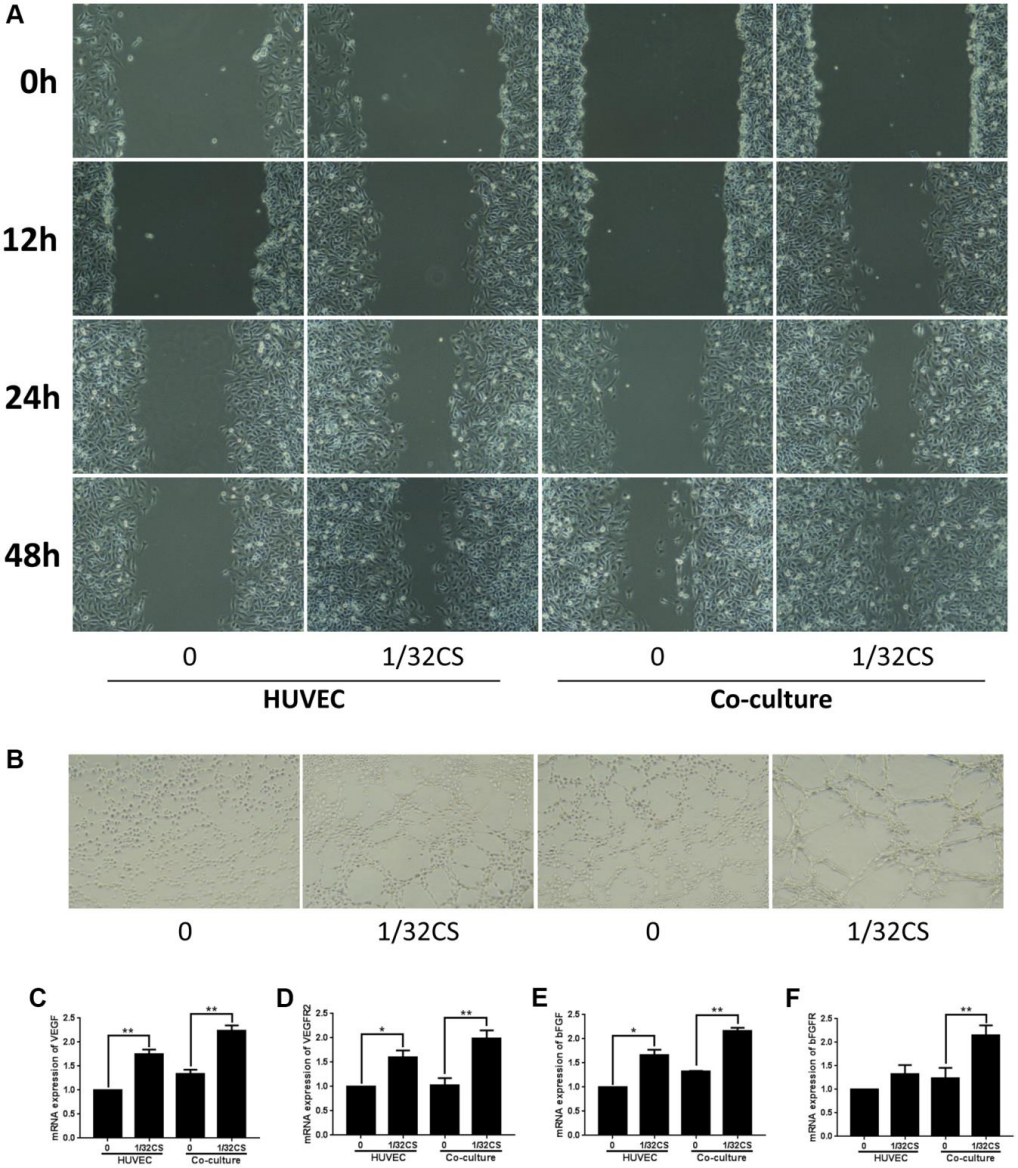
**CS-stimulated ADSCs enhance migration and angiogenic capacity of HUVEC.** (**A**–**F**) The HUVEC was treated with ADSCs, CS, or co-treated with ADSCs and CS. (**A**) The migration was measured by transwell assays in the cells. (**B**) The angiogenic capacity was analyzed by tube formation assays in the cell. (**C**–**F**) The levels of VEGF, VEGFR2, bFGF, and bFGFR were assessed by qPCR in the cells. Data are presented as mean ± SD. Statistic significant differences were indicated: ^*^P < 0.05, ^**^P < 0.01.

To further assess the role of CS-mediated ADSCs in regulating HUVEC phenotypes, the HUVEC was treated with ADSCs or CS, or cotreated with ADSCs and CS. Transwell assays demonstrated that the treatment of ADSCs or CS could induce the migration of HUVEC, in which the co-treated of ADSCs and CS was able to reinforce this effect in the system (Figure 5A). Importantly, the angiogenic capacity was induced by ADSCs or CS in the HUVEC, while the co-stimulation of ADSCs and CS was able to enhance this phenotype (Figure 5B). Moreover, the treatment of ADSCs and CS enhanced the expression of VEGF, VEGFR2, bFGF, and bFGFR in the system (Figure 5C–5F), indicating that CS-stimulated ADSCs enhance migration and angiogenic capacity of HUVEC.

### CS-stimulated ADSCs improve wound healing in full-thickness skin defect mouse model

**Figure 6:**
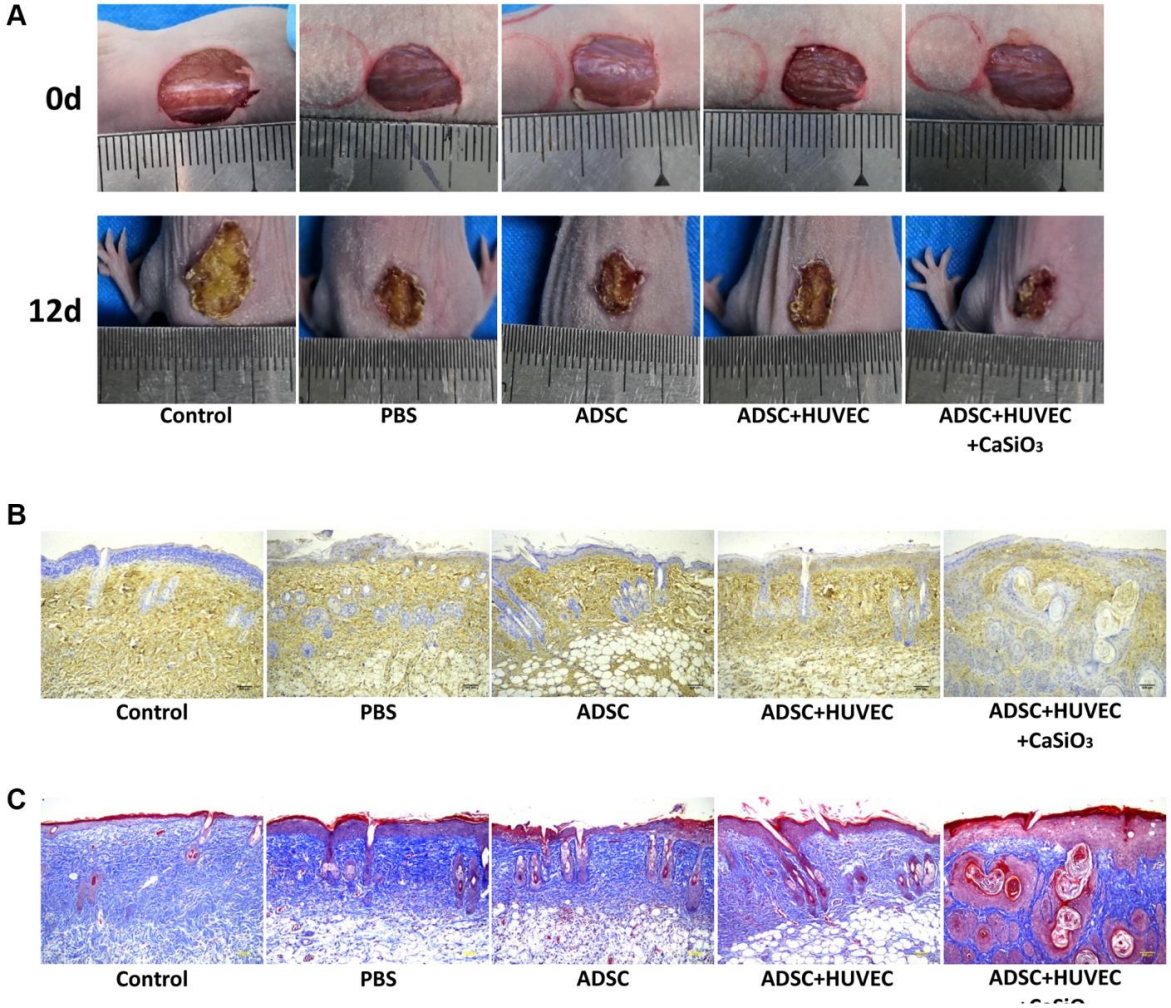
**CS-stimulated ADSCs improve wound healing in full-thickness skin defect mouse model.** (**A**–**C**) A full-thickness skin defect mouse model was constructed, and the mice were treated with ADSCs, ADSCs and HUVEC, or co-treated with ADSCs, HUVEC, and CaSiO3. (**A**) The representative images of wound area were shown. (**B**) The levels of CD31 were measured by immunohistochemistry. (**C**) The collagen deposition was analyzed by Masson’s trichrome staining.

Next, a full-thickness skin defect mouse model was constructed, and the mice were treated with ADSCs, ADSCs and HUVEC, or co-treated with ADSCs, HUVEC, and CS. We identified that the treatment of ADSC could reduce the wound area in the mice, while the co-treatment of HUVEC and CS-stimulated ADSCs could improve the effect in the system (Figure 6A). Besides, the levels of CD31 as an endothelial cell marker were induced by the co-treatment of HUVEC and CS-stimulated ADSCs om the mice (Figure 6B). In addition, Masson’s trichrome staining revealed that the collagen deposition was induced in the system as well (Figure 6C). Together these data suggest that CS-stimulated ADSCs improve wound healing in full-thickness skin defect mouse model.

## DISCUSSION

Wound healing is a prevalent skin disorder with severe injury, in which ADSCs have been identified to play crucial roles. CS presented the potential activities in regulating stem cells and multiple diseases, but the function of CS in ADSCs-mediated Wound healing remains obscure. In this study, we found that CS was able to improve ADSCs-attenuated wound healing *in vivo* and *in vitro*.

CS has demonstrated several biomedical activities in various disease models. It has been reported that CS loaded TiO_2_ fabrication coatings with corrosion resistance and induced biocompatibility through controlling minerals release to improve orthopedic applications [25]. CS microstructure regulates the bioactivity of poly(lactide-co-glycolide) microspheres *in vitro* and *in vivo* [17]. CS stimulate secretion of vascular endothelial growth factor of endothelial cells and mesenchymal stem cell osteogenic differentiation [19]. Meanwhile, the critical function of ADSCs in the modulation of wound healing have been well-recognized. It has been found that adipose improves skin wound healing by promoting the ADSCs differentiation into fibroblasts [26]. The exosomes derived from ADSCs enhance wound healing by PI3K/Akt signaling [27]. ADSCs-derived exosomes up-regulate Nrf2 expression and protect cutaneous wound healing *via* inducing vascularization in the diabetic foot ulcer rat model [28]. ADSCs in combination with Exendin-4 enhances angiogenesis and induces wound healing [22]. In this study, we found that CS is able to enhance the proliferation and migration and can inhibit apoptosis and oxidative stress of ADSCs. Meanwhile, CS-stimulated ADSCs promote migration and angiogenic capacity of HUVEC. Importantly, CS-stimulated ADSCs enhance wound healing in full-thickness skin defect mouse model. Our data demonstrate a critical role of CS in stimulating ADSCs function, presenting informative evidence for the essential function of CS in the ADSCs regulation.

In addition, CXCR4 exerts an important function in the development of ADSCs and wound healing. It has been reported that the elevation of CXCR4 in ADSCs enhances engraftment and homing in the limb ischemia animal model [29]. The CXCR4/CXCR7 axis presents a crucial function the modulation of ADSCs biological behaviors *in vitro* [30]. The delivery to decellularized skin scaffold of CXCR4 antagonist promotes wound healing by inducing migration of CXCR4-positive cells and enhancing SDF-1 expression in diabetic mice [31]. Hair follicle stem cells benefit to cutaneous wound healing by the SDF-1α/CXCR4 signaling [32]. In our investigation, CS can promote CXCR4 expression in ADSCs. It indicates an unreported correlation of CS with CXCR4, implying a new mechanism involving CS and CXCR4 in the modulation of ADSCs.

In summary, we concluded that CS improves ADSCs-attenuated wound healing *in vivo* and *in vitro*. Our finding presents novel insight in the scenario that CS regulates ADSCs and wound healing. CS may be applied as potential materials for the treatment of wound healing.
